# Giant Hydatid Cyst of the Liver Presenting With Severe Chest Pain and Deformity

**DOI:** 10.7759/cureus.45280

**Published:** 2023-09-15

**Authors:** Vugar Suleimanov, Wasayf Almogaliq, Laila O Alibrahim, Fatimah H Alhanabi

**Affiliations:** 1 Surgery, Jubail General Hospital, Jubail, SAU; 2 General Surgery, Imam Abdulrahman Bin Faisal University, Dammam, SAU; 3 Family Medicine, Eastern Health Cluster, Dammam, SAU

**Keywords:** hydatid cyst, echinococcal disease, hydatid cyst of liver, giant hydatid cyst, cystic echinococcosis

## Abstract

Cystic echinococcosis (CE) is a parasitic disease caused by a larval stage (metacestode) of *Echinococcus granulosus* (*E. granulosus*), which is still endemic in many countries worldwide, despite the efforts of the World Health Organization (WHO) to reduce the disease burden in those countries. The hydatid cysts of the liver tend to grow gradually over a long period of time and may cause a variety of symptoms, either related to compression of adjacent organs or rupture. Here we report an unusual case of a giant hydatid cyst of the liver presenting to the emergency room (ER) of our hospital with chest deformity and severe chest pain, heralding impending rupture. Considering the very large size of the cyst (26×20 cm) and severe pain, we deemed expeditious surgery to be the best option. Upon surgery, the cyst contents were evacuated, biliary connections were ligated, and measures were taken to prevent the dissemination of the disease into the abdominal cavity. Closed drainage was used since the patient had almost no omentum. The patient received albendazole pre- and postoperatively. The patient was discharged in good condition after one week in the hospital.

## Introduction

Hydatid cyst, also known as echinococcal disease, is a potentially lethal condition caused by echinococcal larvae and is considered a zoonotic infection. Liver is the most affected organ (70%), followed by the lung (20%), brain, spleen, and kidney [1,2]. It is a highly prevalent disease, especially in endemic areas that include the Middle East [2]. Hydatid cysts might not show any early symptoms since they are slowly growing and can remain asymptomatic for 10-15 years [1]. Hydatid cysts may cause jaundice, abdominal pain, or a visible abdominal mass. Patients usually seek medical attention when the cyst reaches a large size and the liver parenchyma has been destroyed to some degree [3].

The diagnosis is often challenging and requires a combination of clinical examination, imaging, and serology. Magnetic resonance imaging (MRI), computed tomography (CT), and ultrasonography are frequently used imaging modalities for diagnosis. There is a wide variety of radiological imaging findings that are characteristic of the disease. However, these findings can often lead to a wide range of differential diagnoses [4]. The complications of hydatid cysts are rare but can be lethal without early intervention [5]. Intra-biliary rupture of the hydatid cyst is the commonest complication, followed by rupture of the cyst into the peritoneal cavity and thoracic cavity [5]. Surgery remains the mainstay of treatment and should be individualized according to the patient’s overall health status and the predicted perioperative morbidities. In this article, we present the case of an impending rupture of a giant hydatid cyst of the liver and its management.

## Case presentation

**Figure 1:**
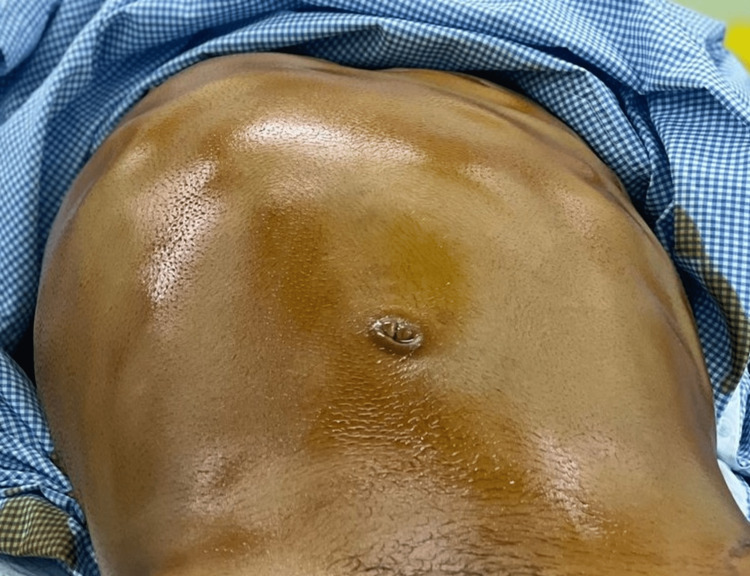
The image on the operating table shows striking deformities of the upper abdomen and chest; the right side of the chest is much larger than the left.

A 26-year-old man presented to the emergency room (ER) of a district general hospital for severe right-sided chest pain of two days duration. The patient had no other symptoms. According to the patient and his companions, the right side of his chest has been enlarging lately. The patient denied any significant past medical or surgical history. He was working as a car mechanic, and there was no contact with animals. The patient had emigrated from Yemen three years ago, which is an endemic region for echinococcosis. On examination, he was in pain, with a heart rate of 112/min, blood pressure of 136/78 mmHg, normal respiratory rate, core body temperature, and oxygen saturation. The right side of his chest was larger than the left side (Figure 1), with no air entry and percussion dullness up to the nipple level.

Left-sided chest exam was normal. There was tenderness over the right subcostal area. Lab tests revealed a high level of alkaline phosphatase and eosinophilia (Table 1). Hydatid serology was requested but was not available in the hospital at the time.

**Table 1:** Laboratory test results. MB: myocardial band

Parameters	Result	Reference range
Creatine kinase MB (CKMB)	30.09 U/L	7-25 U/L
Aspartate aminotransferase (AST)	50 U/L	13-35 U/L
Alkaline phosphatase (ALP)	505 U/L	46-116 U/L
Gamma-glutamyl transferase (GGT)	260 U/L	5-85 U/L
Total bilirubin	26.05 umol/L	3-17 umol/L
Direct bilirubin	9.69 umol/L	0-3.4 umol/L
Serum albumin	31.10 g/L	34-50 g/L
Hemoglobin (Hb)	13.30 g/dL	14-17.4 g/dL
Platelet count (PLT)	311,000/uL	140-410,000/uL
White blood cell count (WBC)	6,900/uL	4-10,000/uL
Eosinophils	9.1%	0-3%

**Figure 2:**
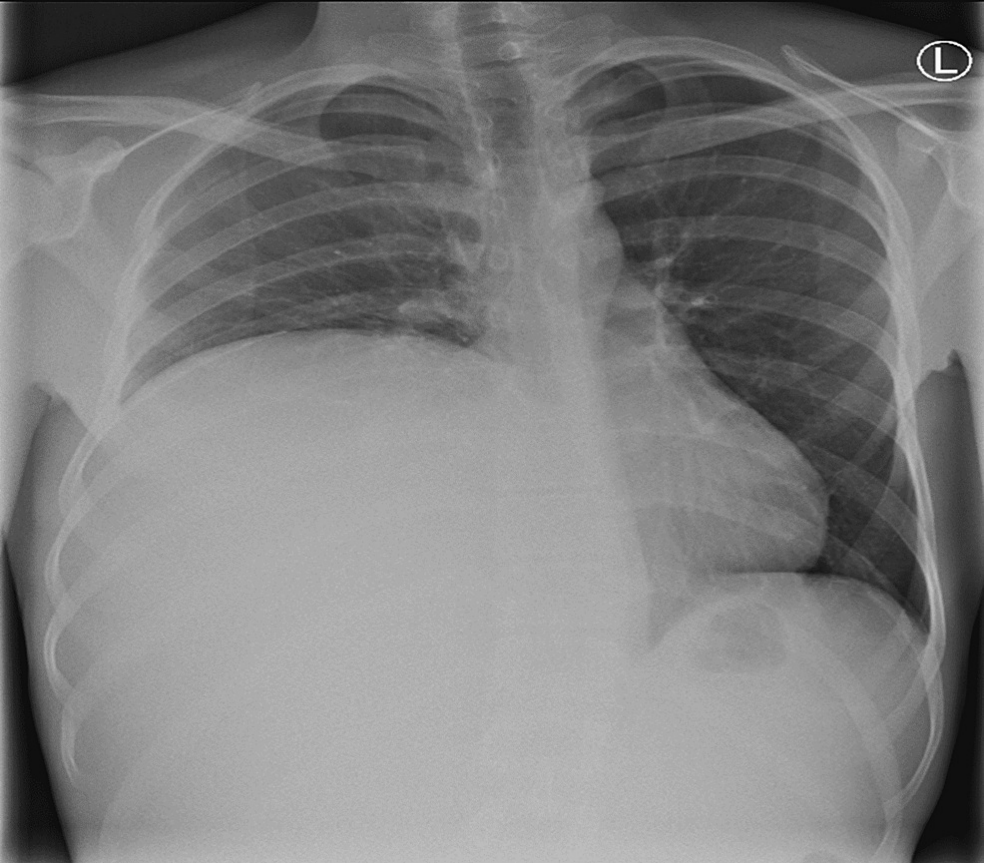
Chest x-ray demonstrates a large liver lesion occupying most of the right side of the chest cavity and displacing mediastinal structures to the left.

A chest x-ray showed upward displacement of the diaphragm (Figure 2).

**Figure 3:**
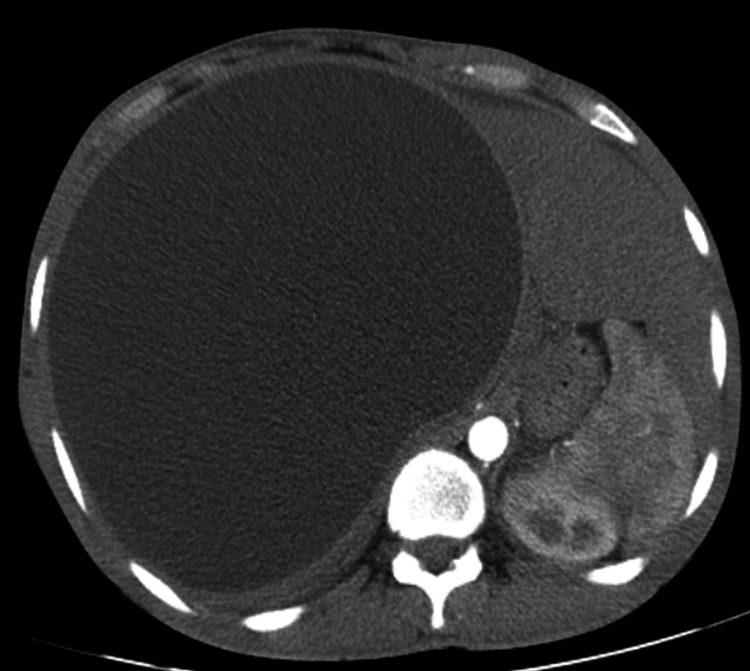
Axial CT scan of the abdomen showing a gigantic liver cyst displacing intra-abdominal organs. CT: computed tomography.

**Figure 4:**
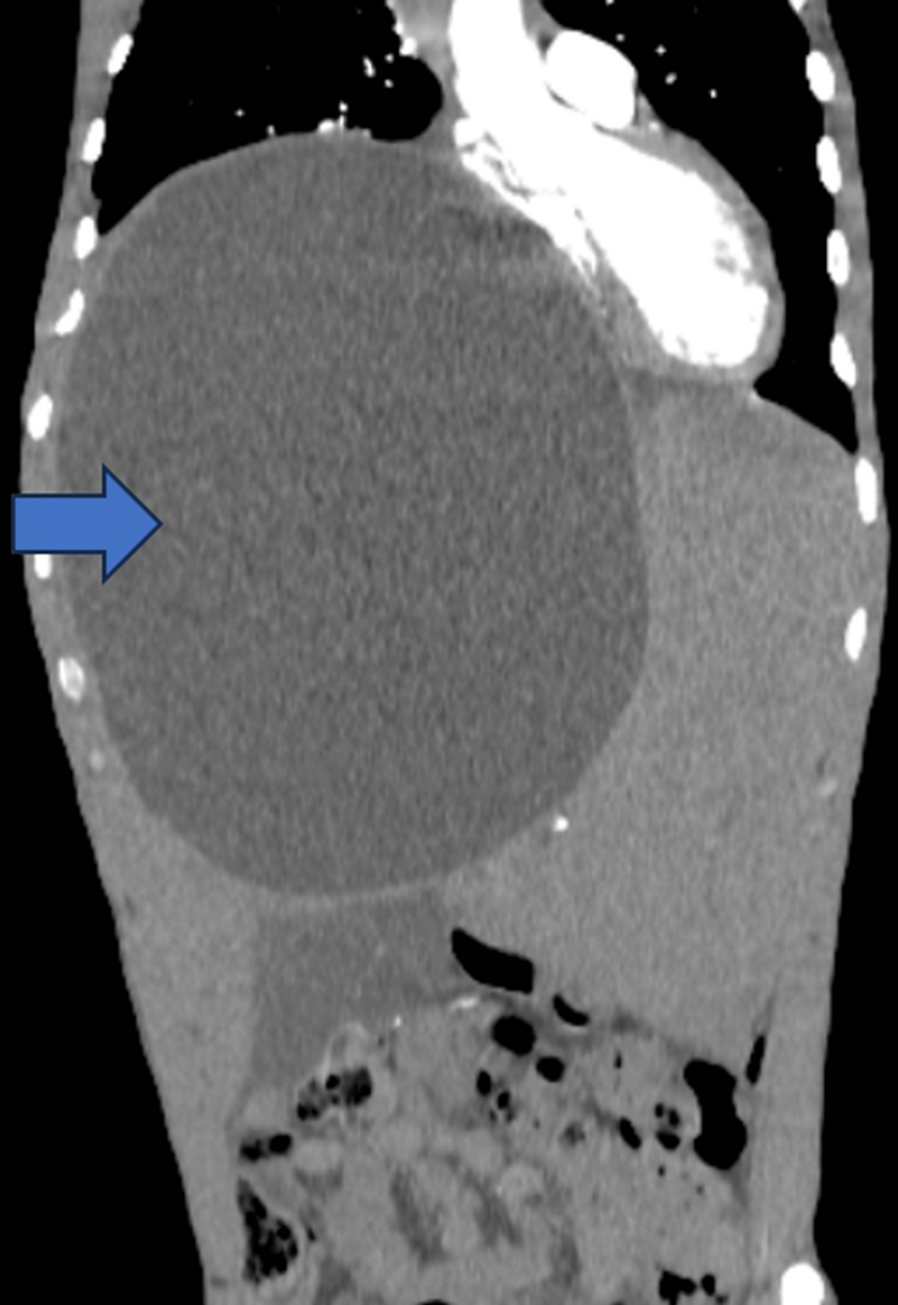
Coronal view of an abdominal CT image showing the cyst (blue arrow) displacing mediastinal structures to the left. CT: computed tomography.

Contrast-enhanced computed tomographic (CECT) images showed a gigantic homogenous cystic lesion of the liver, measuring 26 × 20 cm in its greatest dimensions and significantly displacing abdominal (Figure 3) and intrathoracic organs (Figure 4).

The differential diagnosis included a hydatid cyst as a first possibility, but simple hepatic cysts and biliary cystadenomas were also considered. Since the patient was in severe pain, heralding impending rupture, exploratory laparotomy was chosen after giving the patient two doses of 400 mg albendazole 12 hours apart.

**Figure 5:**
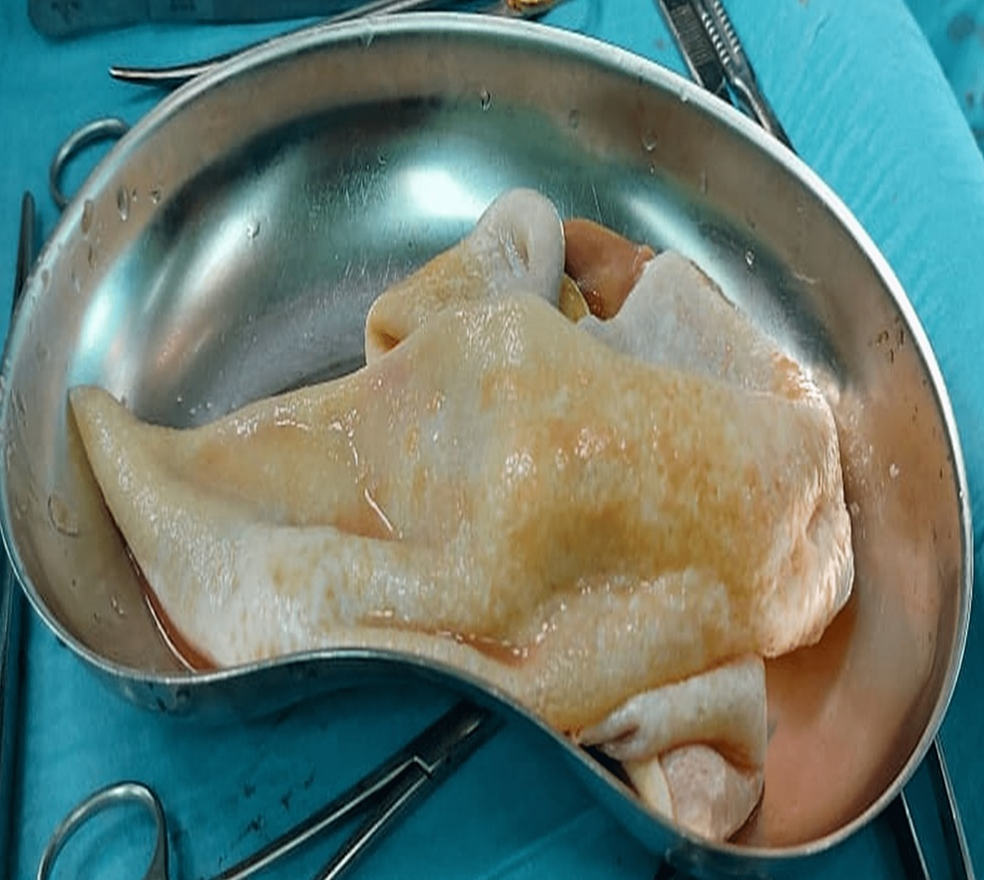
The germinal membrane of the hydatid cyst.

Upon exploratory laparotomy, multiple large abdominal pads soaked with 20% saline were used to isolate the lesion from the rest of the abdominal contents. Hydrocortisone and diphenhydramine injections were kept ready. We initially aspirated the cyst, revealing a clear watery content. After reducing the pressure inside the cyst, we opened the cyst and carefully removed the contents to avoid spillage. At this point, the diagnosis was clear since it contained a typical germinal membrane, which is shown in Figure 5.

**Figure 6:**
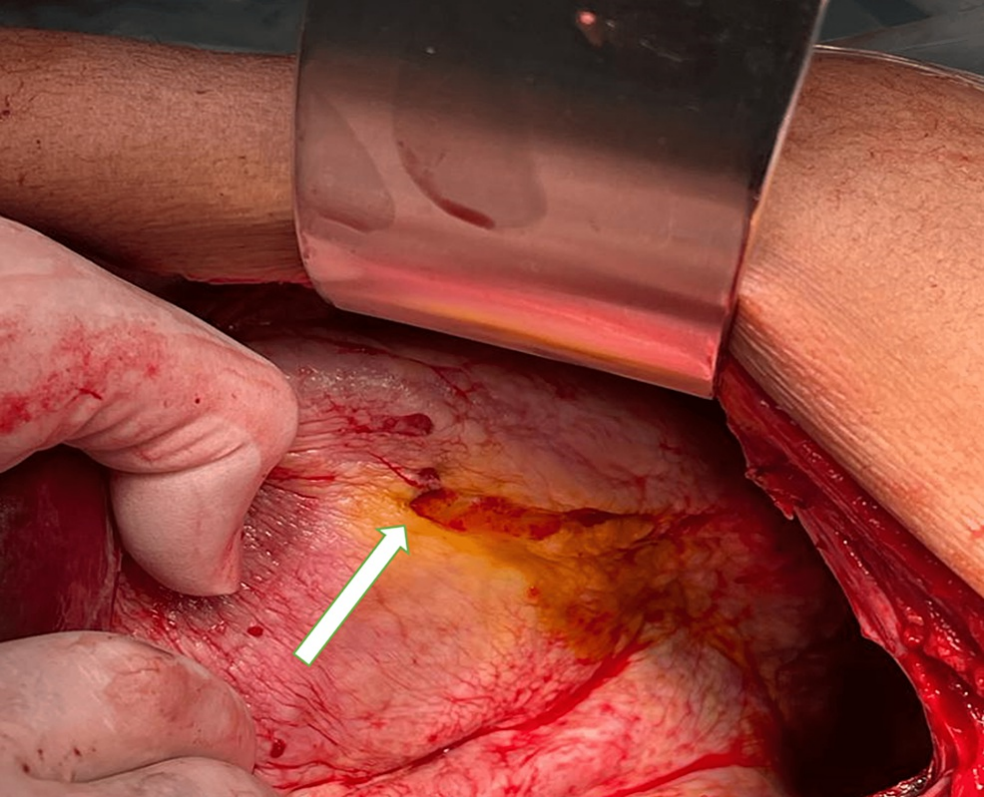
Intraoperative image showing bile leak from a small opening inside the hydatid cyst (white arrow).

Daughter cysts were very small and barely visible to the naked eye. The cyst cavity was repeatedly irrigated with hypertonic saline until clear effluent was observed. Then, the cavity was filled with hypertonic saline and kept for 15 minutes. Before concluding the procedure, the cavity was inspected for biliary communications, and bile leaks were noted at three different points (Figure 6).

Bile leaks from all three openings were stopped by suturing the bile duct openings with 2/0 Vicryl sutures. Omentoplasty was contemplated, but the patient had a very small omentum, so the idea was abandoned. Closed drainage was performed, and the abdominal wound was closed. The patient continued to receive albendazole postoperatively; he had a smooth recovery without any complications. The patient weighed 45 kg postoperatively (preoperative weight was 61 kg), so we assume the cyst weighed 16 kg. After one week of admission, the patient was discharged home to be followed up as an outpatient.

On follow-up at one month, six months, and one year, the patient was asymptomatic, gained weight, and had no recurrence.

## Discussion

Hippocrates is believed to have described human hydatid disease about a couple of millennia ago [6]. It has been considered a neglected public health problem in developing nations. The sheep-raising industry and dogs have been significantly related to the development of hydatid cyst disease, as reported in the literature; however, high clinical suspicion is required despite the absence of an exposure history to infected animals [7,8]. Given that 75% of hydatid cysts are in the liver, studies have found higher involvement of the right liver lobe due to high blood supply to the right lobe compared to the left lobe, as described by Maingot, which is congruent with our case [9,10].

Clinically, a hydatid cyst presents clinically according to the size, location, and depth of the cyst. In earlier phases, patients might be asymptomatic when the cyst is small. As the condition progresses, an enlarging liver hydatid cyst eventually manifests as epigastric or right upper quadrant abdominal discomfort, nausea, vomiting, and hepatomegaly. Patients might present with complications due to rupture, secondary bacterial infection, and effects on adjacent structures [11]. Rarely, the cyst may reach an enormous size without bothering the patient, like in our case, where a huge liver cyst was found to be occupying most of the right side of the chest and abdominal cavity. On reviewing the literature, giant hydatid cysts of the liver may rarely rupture, leading to fatal consequences such as anaphylactic shock [1]. Therefore, treatment of giant hydatid cysts should not be delayed. Cyst diameter >10 cm, young age, and superficial cyst site are the main risk factors predisposing to rupture, as recognized in our case [12, 13].

A complete approach to hydatid cysts is carried out through a clinical and radiological assessment, laboratory, serological tests, and histopathological evaluation. Elevated eosinophil count may point toward the presence of parasitic infections, as in our patient. Enzyme-linked immunosorbent assay (ELISA) and indirect hemagglutination tests have a sensitivity of 80% for echinococcosis [14,15]. However, both tests were not available in our hospital.

The therapeutic approach to hepatic hydatid disease encompasses medical, surgical, and endoscopic interventional treatments [16]. Some experts recommend a watch-and-wait approach for inactive cysts [17]. Albendazole and mebendazole are considered effective chemotherapy for hydatid cysts, especially small and multiple cysts, inoperable hydatid cysts and three to six months duration is considered to be adequate if medical management is chosen [16]. Percutaneous management, like puncture, aspiration, injection, and re-aspiration (PAIR) techniques, has been used for eligible cases for more than three decades with good outcomes, though it is recommended to rule out cysto-biliary fistulae before injecting scolecidal agents into the cyst cavity [17].

The Gharbi classification is the most commonly used classification for hydatid cyst management, which evaluates the cyst ultrasonographically. According to this classification, all cysts should be excised surgically or treated with the PAIR technique, except the small ones [1]. Surgical resection is considered the cornerstone for large hydatid cysts of the liver, specifically in patients where cysts cause significant compression of adjacent structures due to their enormous size, as seen in our case [16].

Regarding surgical management, although it has been replaced with less invasive treatment modalities in recent years, there are some instances where surgery is the best option, especially for complications of the cyst (rupture, cysto-biliary fistula, compression of vital structures, superinfection, hemorrhage). Surgery is considered the best option for large cysts at high risk of rupture, like in our case, or cysts that are not amenable for percutaneous treatment modalities [16].

The surgical approach can be radical or conservative. If radical surgery is undertaken, either pericystectomy or liver resection is carried out, depending on the size of the cyst, but giant hydatid cysts are usually not amenable for radical resection, like in our case. On the other hand, conservative surgery refers to the removal of the germinal membrane along with the cyst contents and sterilization of the residual cavity with one of the scolicidal agents, together with partial cyst resection [18]. In our patient, the giant cyst was densely adherent to the diaphragm and mediastinal structures, and the size was very huge, so an attempt at radical surgery would be counterproductive, which is why we chose a conservative approach.

## Conclusions

Even though there is no consensus about the optimal management of giant hydatid cysts, most authors recommend a surgical approach to avoid serious morbidity and even mortality. When an otherwise asymptomatic patient with a giant hydatid cyst of the liver or any other site presents with severe pain, it heralds an impending rupture, and we recommend an expeditious surgical approach to prevent anaphylactic shock, death, or other complications.
